# Applying Data Mining Techniques to Extract Hidden Patterns about Breast Cancer Survival in an Iranian Cohort Study 

**Published:** 2015-03-21

**Authors:** Hamid Reza Khalkhali, Hadi Lotfnezhad Afshar, Omid Esnaashar, Nasrollah Jabbari

**Affiliations:** ^a^ Inpatient’s Safety Research Center, Department of Biostatistics, School of Medicine, Urmia University of Medical Sciences, Urmia, Iran; ^b^ Department of Health Information Technology, School of Paramedicine, Urmia University of Medical Sciences, Urmia, Iran; ^c^ Omid Treatment and Research Center, Urmia, Iran; ^d^ Solid Tumor Research Center, Department of Medical Physics and Imaging, School of Paramedicine, Urmia University of Medical Sciences, Urmia, Iran

**Keywords:** Breast Neoplasms, Survival, Data Mining, CART, Decision Tree

## Abstract

**Background:** Breast cancer survival has been analyzed by many standard data mining algorithms. A group of these algorithms belonged to the decision tree category. Ability of the decision tree algorithms in terms of visualizing and formulating of hidden patterns among study variables were main reasons to apply an algorithm from the decision tree category in the current study that has not studied already.

**Methods:** The classification and regression trees (CART) was applied to a breast cancer database contained information on569 patients in 2007-2010. The measurement of Gini impurity used for categorical target variables was utilized. The classification error that is a function of tree size was measured by 10-fold cross-validation experiments. The performance of created model was evaluated by the criteria as accuracy, sensitivity and specificity.

**Results:** The CART model produced a decision tree with 17 nodes, 9 of which were associated with a set of rules. The rules were meaningful clinically. They showed in the if-then format that Stage was the most important variable for predicting breast cancer survival. The scores of accuracy, sensitivity and specificity were: 80.3%, 93.5% and 53%, respectively.

**Conclusions:** The current study model as the first one created by the CART was able to extract useful hidden rules from a relatively small size dataset.

## Introduction

Survival prediction of cancers is an important field in data mining domain^1^. Prediction of breast cancer survival is a major area of interest within this domain because breast cancer is the second leading cause of cancer death and the most commonly diagnosed cancer after skin cancer^2-3^.

Breast cancer survival has been analyzed with the various data mining methods. Decision tree algorithms belonged to classification category of data mining have been studied for predicting breast cancer survival^1,4-10^. Researchers have used the decision tree algorithms such as: C5 or J48, ID3 and CHi-squared Automatic Interaction Detection (CHAID) along with other data mining classification methods to predict survival status of breast cancer patients 60 months after diagnosis. All of them but one^6^ have evaluated the Surveillance Epidemiology and End Results (SEER) dataset. The SEER is a dataset that provides socio-demographic and cancer specific information about the U.S. population^11^. The visualization of decision trees and formulating them into if-then rules easily can be done^12^. The analysis of built tree and produced rules from it lead to a more clear understanding of hidden pat-terns and relations between dataset variables. The built tree and its rules only have been reported and analyzed by‏ Wang et al.^8^.

A model developed by classification and regression trees (CART) for breast cancer survival prediction applied in the regional dataset originated from a Middle East countryis not described in the literature. The CART is a recursive partitioning method^13^ primarily used as a classification tool. It recursively splits the training data into a set of segments with similar outcome variable values. CART works by examining the predictor variables to find the best split in each segment. All splits in each partitioning step are binary and splitting process starts by defining two subgroups, and so on, until one of the stopping criteria is met. Because the dataset in the present study primarily contained categorical data, CART analysis was chosen as the research methodology from among the many classification tree models.

The objective of this study was to employ data mining to identify hidden pattern and important rules crucial to the survival status of breast cancer in an Iranian’s medical research center between 2007 and 2010.

## Methods

### Context and Data Source

Data were obtained from the Omid Treatment and Research Center, a charity organization for supporting cancer patients of West Azerbaijan in Iran. The center has averagely 7,000 annual admissions. Service lines include: specialized clinic for cancer, radiotherapy ward equipped with two medical linear accelerator machines, brachytherapy equipment, simulator and CT simulator, chemotherapy, specific medical physics wards, mammography and dentistry, ophthalmology and psychology clinics.

After consulting with oncologists of center and studying domain literature^1,4-10^, the important variables were extracted from paper medical records of female patients with breast cancer. The source of information about final status of patients (Alive/Dead) was from their medical record or through call phones.

### Study Populations

This study was a cohort one. The dataset analyzed in this study included demographics, therapeutic and survival information from 569 patients (mean age 48.6 yr, standard deviation 10.6) between the years 2007 and 2010. All patients who did not meet exclusion criteria were included to avoid potential sampling bias. Patients excluded were: male, followed up less than sixty months and still alive, and followed up less than sixty months and the cause of their death was not breast cancer.

### Data Processing

For detecting the noisy data (missing and outliers) and developing the model, data were entered to IBM SPSS Statistics 22 (Chicago, IL, USA). The noisy data have negative effect on the model performance^14^. No outlier was in this dataset, but some variables had missing values. The missing data proportion in variables is showed in Table 1.The most common method to handle missing data is deleting of them^15^.

**Table 1:** The proportion of missing data

**Variable Name**	**Number**	**Percent**
Primary site of tumor	30	5.3
Histology of tumor	28	4.9
Tumor Size	31	5.4
Metastases of tumor	27	4.7
Stage of tumor	26	4.6
Behavior of tumor	22	3.9
Grade of tumor	35	6.2
Positive regional nodes	29	5.1
Removed regional nodes	37	6.5
Surgery of tumor	4	0.7
Human epidermal growth factor receptor 2(Her2)	131	23.0
Estrogen receptor	65	11.4
Progesterone receptor	65	11.4
Survival	5	0.9

**Figure 1:**
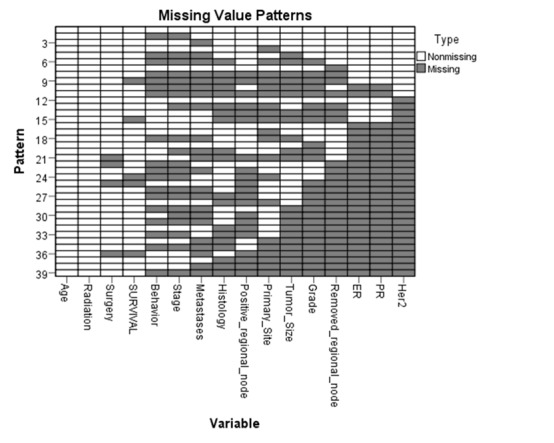
Pattern of missing data

Dataset used in this study was not large, so deleting missing data decreased the size of dataset. To prevent data loss, the missing data were imputed by the multiple imputation (MI) method. Missing data are imputed by multiple values in this method. Because exact estimation of missing data is scientifically impossible, multiple values are generated to manage this uncertainty^16^. To do MI method in IBM SPSS Statistics 22, the pattern of missing data was analyzed before imputation. Figure 1 shows missing value patterns for the analysis variables. Each pattern corresponds to a group of cases with the same pattern of incomplete and complete data. For example, Pattern 1 represents cases which have no missing values, while Pattern 2 represents cases that have missing values on Behavior of tumor and Stage of tumor variables. This figure orders analysis variables and patterns to reveal monotonicity where it exists. If the data are monotone, then all missing cells and non-missing cells in the figure will be contiguous; that is, there will be no “islands” of non-missing cells in the lower right portion of the chart and no “islands” of missing cells in the upper left portion of the chart^17^. Dataset used in this study is monotone. The monotone method fits a univariate (single dependent variable) model using all preceding variables in the model as predictors, then imputes missing values for the variable being fit. These imputed values are saved to the imputed dataset^17^. The analysis of missing patterns revealed that MI could be done on our dataset. After implementation MI, five complete datasets were created.

### Model development

Table 2 shows the variables used in developing the model and their descriptive statistics after MI. There are 15 predictor variables and 1 outcome variable (Survival). The outcome variable shows survival status of patients in the specified period of time (60 months) after diagnosis.

**Table 2:** Descriptive statistics of study variables

**Categorical variables**	**Number**	**Percent**
Primary site of tumor		
Central	104	18.3
Upper inner quadrant	217	38.1
Lower inner quadrant	83	14.6
Upper outer quadrant	112	19.7
Lower outer quadrant	53	9.3
Metastases of tumor		
Yes	158	27.8
No	411	72.2
Behavior of tumor		
In situ	208	36.6
Malignant	361	63.4
Grade of tumor		
I	187	32.9
II	302	53.1
III	80	14.1
Histology of tumor		
Adenoid	6	1.1
Ductal carcinoma in situ	17	3.0
Epithelial	3	0.5
Invasive ductal carcinoma	486	85.4
Paget disease	7	1.2
Comedo	6	1.1
Invasive lobular carcinoma	15	2.6
Inflammatory	2	0.4
Mucinous	5	0.9
Papillary	5	0.9
Micropapillary	5	0.9
Medullary	10	1.8
Phyllodes	2	0.4
Stage of tumor		
1	18	3.2
2A	123	21.6
2B	102	17.9
3A	151	26.5
3C	26	4.6
4	149	26.2
Surgery of tumor		
No	109	19.2
Lumpectomy	2	0.4
Quadrantectomy	7	1.2
Modified radical mastectomy	447	78.6
Radical mastectomy	4	0.7
Radiation of tumor		
Yes	506	88.9
No	63	11.1
Human epidermal growth factor receptor 2 (Her2)		
Yes	255	44.8
No	314	55.2
Estrogen receptor		
Yes	261	45.9
No	308	54.1
Progesterone receptor		
Yes	288	50.6
No	281	49.4
Survival		
Yes	384	67.5
No	185	32.5
**Continuous variables**	**Mean**	**SD**
Age	48.6	10.6
Tumor size	4.0	1.9
Number of positive regional nodes	3.6	4.3
Number of removed regional nodes	8.4	5.7

For the purpose of study, CART was used for model development. CART uses a binary recursive process. This process is started by splitting subsets of the complete dataset (using all predictor variables) to two child nodes repeatedly. A variety of impurity or diversity measures (Gini, twoing, and ordered twoing) are used for choosing of the best predictor. The produced subsets of the data should be as homogeneous as possible with respect to the target variable. In this study, the measurement of Gini impurity that used for categorical target variables was used. The classification error that is a function of tree size was measured by 10-fold cross-validation experiments. The dataset were randomly split into 10 smaller subsets. Choosing of the best number of nodes from the original tree was performed by a backward pruning method. After building the 10 trees, their classification error rate was averaged and the tree producing the least amount of misclassification was selected as the optimal tree.

IBM SPSS Statistics 22 was used for running CART. This algorithm was applied to five complete datasets created from MI method. Model with the highest evaluation criteria was selected as final model.

### Model evaluation

The performance estimation of model was evaluated by 3 criteria: sensitivity, specificity and accuracy. The formulas of these criteria are:

$$\begin{array}{l} Accuracy=\frac{TP+TN}{TP+TN+FP+FN}=\frac{true\;positive+true\;negative}{true\;positive+true\;negative+false\;positive+false\;negative}\\ Sensitivity=\frac{TP}{TP+FN}=\frac{true\;positive}{true\;positive+false\;negative}\\ Accuracy=\frac{TN}{FP+TN}=\frac{true\;negative}{false\;positive+true\;negative} \end{array}$$

## Results

**Figure 2:**
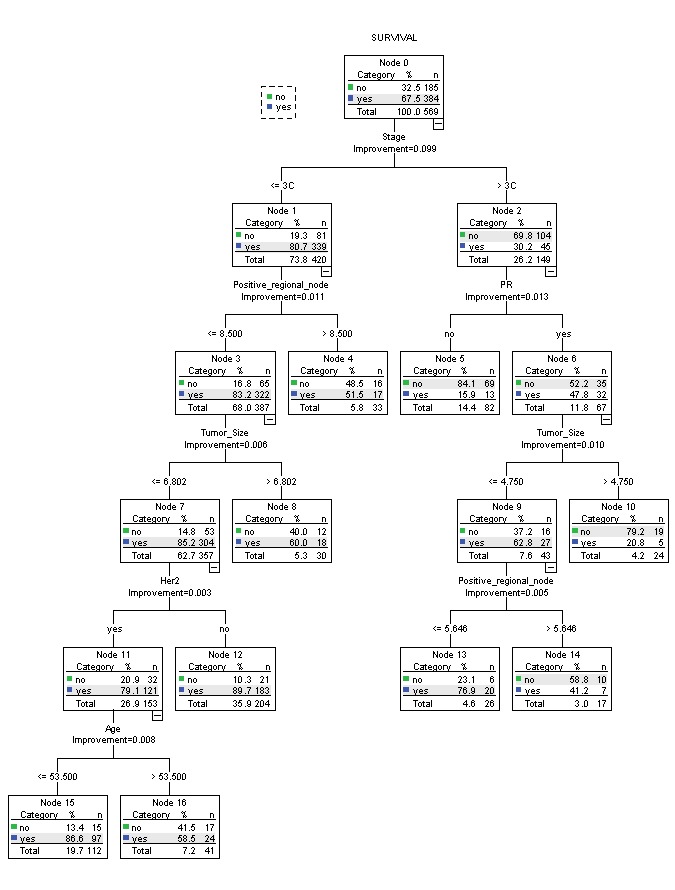
Extracted hidden pattern by classification and regression trees (CART)

CART algorithm was used to extract hidden pattern in the breast cancer dataset. Prior to CART analysis, the dataset was divided to training and testing data. The decision tree was built from training data, and its predictive accuracy was tested by applying it to predict the class label (Survival values in this case). Figure2 displays the decision tree of CART classifying the survival status of breast cancer.

The resulted decision tree has 17 nodes generally and 9 leaf nodes and each leaf node is associated with a set of rules. Table 3 summarizes the rules for each category of survival status from the tree. The rules have been evaluated and approved by the oncologists for an appropriate survival classification.

The rules have been arranged in Table 3 based on sensitivity scores. As shown in Figure 2 and appeared from rules, Stage was the most important predictor because it was at the top node of the decision tree. An example of the rules for leaf node 16 can be read as: “IF value of Stage was less than or equal to 3C, AND value of Positive-regional-node was less than or equal to 9, AND value of Tumor size was less than or equal to 7, AND value of Human epidermal growth factor receptor 2 (Her2) was equal to yes, AND value of Age was greater than 54, THEN the predicted survival status belonged to Class yes.”Overall sensitivity, specificity and accuracy of model were: 93.5%, 53% and 80.3% respectively.

**Table 3:** Extracted rules for each category of survival status

**Leaf node**	**Rule number**	**Rules content**	**Sensitivity (%)**
Survival status (Yes)
12	1	If Stage ≤3C & Positive-regional-node ≤9 & Tumor size ≤7 & Her2 = No	89.7
15	2	If Stage ≤3C & Positive-regional-node ≤9 & Tumor size ≤7 & Her2 = Yes & Age ≤54	86.6
13	3	If Stage >3C & PR = Yes & Tumor size ≤5 & Positive-regional-node ≤6	76.9
8	4	If Stage ≤3C & Positive-regional-node ≤9 & Tumor size >7	60.0
16	5	If Stage ≤3C & Positive-regional-node ≤9 & Tumor size ≤7 & Her2 = Yes & Age >54	58.5
4	6	If Stage ≤3C & Positive-regional-node >9	51.5
Survival status (No)
5	1	If Stage >3C & PR = No	84.1
10	2	If Stage >3C & PR = Yes & Tumor size >5	79.2
14	3	If Stage >3C & PR = Yes & Tumor size ≤5 & Positive-regional-node >6	58.8

## Discussion

The current study found that variable of Stage was the most important predictor to predict breast cancer survival status. However, this result has not previously been described. Variable of Positive-regional-node was the most important predictor to predict breast cancer survival status^8^. In the current study, this variable was the most important variable after Stage variable. This inconsistency may be due to nature of the used algorithms or databases. Although, discrepancy existed between the most important variables, but there were some consistencies between variables of extracted important rules. The variables: Stage, Positive-regional-node, Tumor size and Her2 constituted the first rule of current study that had highest sensitivity (89.7%). Three of those variables (Stage, Positive-regional-node and Tumor size) were also in the first rule of Wang KJ’s et al. study^9^. In the current study, Her2 was one of important variables for predicting survival status. HER2 (human epidermal growth factor receptor 2) is one such gene that can play a role in the development of breast cancer^18^. This variable has not been reported in the SEER dataset^11^, so paper of Wang et al. had no information about it^9^.

The Wang et al. study was more imbalanced than current study. That means that distribution of class variable (Survival variable) was not balanced. In other words one class had overwhelming number of instances than the other class. In such situations the performance of most standard data mining algorithms are with bias and do not reflect real results^6^. Distribution of class variable in their paper was 90.68% (Survival) to 9.32% (Non-survival). This distribution in current study was 67.5% (Survival) to 32.5% (Non-survival). They had to use synthetic minority oversampling technique (SMOTE) to manage this challenge.

The imputation of missing data has not been done in the studies on breast cancer survival performed on the SEER dataset^1,4-9^, except one^10^. Because the SEER dataset is a large one, the authors of mentioned papers may have preferred to delete missing data and not impute them. In current study, imputation of missing data has prevented the remarkable loss of information about breast cancer survival status. The other reason for doing imputation of missing data in this study in addition to keeping valuable information was small size of used dataset. The applied technique in Afshar et al. work^10^ and current study was the same. In both studies MI was used.

The sensitivity of model created in our study (93.5%) was higher than sensitivity of Ahmadi et al. ^10^, Wang et al.^8^ and Thongkam et al.^6^ models. The sensitivity of Ya-Qin‏ et al.^7^, Delen et al.^1^, Endo et al.^5^ and Wang et al^9^ were higher than current studies’ sensitivity. This criterion has not been reported in Bellaachia and Guven^4^ study. Among studies performed onbreast cancer survival, the specificity of current study (53%) was higher than Ya-Qin‏ et al^7^ and Endo et al.^5^ works. The Bellaachia and Guven^4^ has not reported this criterion like as sensitivity. Based on mentioned results, it is clear that our model predicted survived status better than non-survived status. The accuracy of all studies except Wang et al.^8^ study was higher than our study’s (80.3%). Although, the evaluation criteria: accuracy and specificity were lower than the most studies, but it is not a limitation for the current study because the evaluation criteria: accuracy, sensitivity and specificity are not unquestioning criteria to judge about a model performance.

One limitation of our research was the database size. Because current study’s dataset was small, the training data of it was not large too. There is a direct relationship between learning power of data mining algorithms and number of training data^12,19^. Another limitation of current research was the percentage of missing values of Her2. Although Her2 was only variable in our study and missing data of it were above 20 percent, besides, its missing data were substituted by a suitable method.

## Conclusions

The main goal of the current study was to create a model by a data mining algorithm to extract important pattern and rules from a regional dataset about breast cancer survival data. The main result of this study showed that variable of Stage was the most important predictor for predicting breast cancer survival. Another result of current study showed that the created model has a tendency to predict status of patients that have higher probability to survive from breast cancer better than patients that have lower probability to survive. This is the first study that used the CART in a regional database. This algorithm has not been used in previous studies on breast cancer survival. Ongoing study is needed to apply CART to a dataset with more records and dataset that has contained information about patients followed up 10 or 15 yrafter cancer diagnosis.

## Acknowledgments

The authors acknowledge the Vice Chancellor of Research and Technology, Urmia University of Medical Sciences, which approved this project, and thank all the staffs and directors in Omid Treatment and Research Center.

## Conflict of interest statement

The authors have no conflicts of interest to declare for this study.
